# Half‐life extension of single‐domain antibody–drug conjugates by albumin binding moiety enhances antitumor efficacy

**DOI:** 10.1002/mco2.557

**Published:** 2024-05-09

**Authors:** Quanxiao Li, Yu Kong, Yuxuan Zhong, Ailing Huang, Tianlei Ying, Yanling Wu

**Affiliations:** ^1^ MOE/NHC/CAMS Key Laboratory of Medical Molecular Virology Shanghai Institute of Infectious Disease and Biosecurity Shanghai Frontiers Science Center of Pathogenic Microorganisms and Infection Shanghai Engineering Research Center for Synthetic Immunology Department of medical microbiology and parasitology, School of Basic Medical Sciences Fudan University Shanghai China; ^2^ College of Life Sciences Hebei Agricultural University Baoding China

**Keywords:** albumin‐binding moiety, antitumor efficacy, half‐life extension, single‐domain antibody–drug conjugates

## Abstract

Single‐domain antibody–drug conjugates (sdADCs) have been proven to have deeper solid tumor penetration and intratumor accumulation capabilities due to their smaller size compared with traditional IgG format ADCs. However, one of the key challenges for improving clinical outcomes of sdADCs is their abbreviated in vivo half‐life. In this study, we innovatively fused an antihuman serum albumin (αHSA) nanobody to a sdADCs targeting oncofetal antigen 5T4, conferring serum albumin binding to enhance the pharmacokinetic profiles of sdADCs. The fusion protein was conjugated with monomethyl auristatin E (MMAE) at s224c site mutation. The conjugate exhibited potent cytotoxicity against various tumor cells. Compared with the nonalbumin‐binding counterparts, the conjugate exhibited a 10‐fold extended half‐life in wild‐type mice and fivefold prolonged serum half‐life in BxPC‐3 xenograft tumor models as well as enhanced tumor accumulation and retention in mice. Consequently, n501–αHSA–MMAE showed potent antitumor effects, which were comparable to n501–MMAE in pancreatic cancer BxPC‐3 xenograft tumor models; however, in human ovarian teratoma PA‐1 xenograft tumor models, n501–αHSA–MMAE significantly improved antitumor efficacy. Moreover, the conjugate showed mitigated hepatotoxicity. In summary, our results suggested that fusion to albumin‐binding moiety as a viable strategy can enhance the therapeutic potential of sdADCs through optimized pharmacokinetics.

## Graphical abstract

We innovatively fused an antihuman serum albumin nanobody to a single‐domain antibody–drug conjugates targeting oncofetal antigen 5T4, conferring serum albumin binding, exhibiting a 10‐fold extended half‐life and enhanced tumor accumulation and retention in mice. Consequently, it demonstrated significantly improved antitumor efficacy in tumor xenografts despite less frequent dosing with mitigated hepatotoxicity.

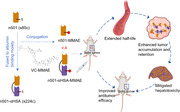

## 1 INTRODUCTION

Over the past 20 years, antibody–drug conjugates (ADCs) have demonstrated promising clinical efficacy in treating various tumors.^1^ The key components of an ADC include the antibody, the cytotoxic payload, and the linker that connects the antibody to the payload.^2^, ^3^ Upon binding to tumor surface‐associated antigens, they are internalized and undergo lysosomal degradation, releasing small molecule toxins that damage tumor cells, typically DNA damaging agents or protease inhibitors, which enhanced targeting specificity and safety.^3^

Due to the formation of a “binding site barrier” in solid tumors, traditional ADCs penetrate only a few cell layers beyond blood vessels, leading to ADC accumulation near blood vessels and difficulty in penetrating the tumor interior.^4^ In contrast, small molecule antibody ADCs have faster diffusion coefficients, allowing them to penetrate tissues further before binding to their targets. In recent years, to enhance tumor penetration and intratumoral diffusion of ADCs, full‐length antibodies are being replaced with smaller yet high‐affinity binders, such as peptides and single‐domain antibodies.^5^, ^6^ These small‐sized binders are developing rapidly due to their advantages such as easy synthesis or expression, low production cost, and low immunogenicity, especially these small‐sized binders can permeate into tumor tissues and improve the exposure of the conjugated payload, thereby boosting therapeutic effect, especially in treating solid tumors.^7^ However, it still has some challenges, such as peptide–drug conjugates (PDCs), which have poor stability and have weaker targeting capabilities compared with IgG‐format ADCs.^8^, ^9^ In addition, due to the higher affinity binding with the antigen and the small size of the nanobody, it is easier to bind to hidden epitopes and has high stability,^10^ which has attracted much attention. However, because it comes from camels or sharks, which has certain immunogenicity.^11^ Thus, there is an urgent need to identify highly stable, high‐affinity, low‐molecular weight and lower immunogenicity proteins for solid tumor therapy.

Our group solved this problem by grafting the human antibody heavy chain variable domain into the framework region of a fully human germline gene to constructing a single domain antibody library and obtaining human‐derived single domain antibody.^12^ However, the common challenge of small‐sized binder is that the clinical application of these smaller biotherapeutics is often hindered by their rapid clearance and the consequent short circulating half‐life.^13^ High dosing frequency and dose levels are required to maintain therapeutic concentrations, posing challenges in administration convenience and patient compliance.

To mitigate the rapid clearance limitation of small‐size therapeutics, diverse half‐life extension strategies have been explored over the last decades. Notable approaches include fusion with the Fc domain of human IgG, PEGylation, and fusion to human serum albumin (HSA) or albumin‐binding moieties, which have exhibited clinical promise in augmenting the systemic retention and exposure of small proteins and peptides.^14^, ^15^, ^16^, ^17^, ^18^, ^19^, ^20^ HSA is the most abundant protein in plasma (∼40 g/L), with wide tissue distribution, high stability, solubility, and a half‐life of approximately 19 days.^21^, ^22^ HSA utilizes the neonatal Fc receptor (FcRn)‐mediated recycling to extend the pharmacokinetics (PK) of fused proteins.^16^ Fusion to HSA or albumin‐binding moieties has effectively extended the half‐life of various small‐size therapeutics.^23^ For instance, ozoralizumab, a drug approved for rheumatoid arthritis, consists of two nanobodies targeting TNF‐α and one nanobody targeting human HSA, thereby extending the half‐life of the 38 kDa drug to a *t*_1/2_ of 23 h in mice.^24^ Furthermore, albumin accumulation in solid tumors due to leaky vasculature and impaired lymphatic drainage could confer particular advantages to HSA‐based strategies for cancer therapy.^25^ Consequently, half‐life extension approaches utilizing HSA have attracted increasing attention.^26^ Despite these advances, the aforementioned half‐life extension strategies have not yet been extensively explored for application to ADCs to improve PK and enhance antitumor efficacy.

Our group has developed a fully human single‐domain antibody targeting the oncofetal antigen 5T4 conjugated to SN38 (n501‐SN38), which demonstrated stronger tumor penetration and faster tumor accumulation compared with full‐length IgG1 format ADC, resulting in improved antitumor efficacy.^12^ In order to extend the half‐life of n501–drug conjugate and investigate whether half‐life extension can improve the therapeutic potential of small‐sized ADCs, we engineered n501 by fusing it with an anti‐HSA (αHSA) nanobody, designated n501–αHSA. In previous studies we found that the hydrophobicity of SN38 would cause n501–s85c to aggregate and precipitate during the conjugation, thus increasing production costs, so we chose MMAE, the most widely used small molecule toxin in approved ADCs, exploring half‐life extension of n501–MMAE. Fusion with αHSA led to over 10‐fold extension in half‐life. This ADC (n501–αHSA–MMAE) showed potent cytotoxicity against 5T4^+^ tumor cells in vitro and in vivo. Furthermore, compared with n501–MMAE, n501–αHSA–MMAE showed reduced liver and kidney accumulation coupled with enhanced intratumoral accumulation. More importantly, the extended circulation time and increased tumor accumulation resulted in superior antitumor efficacy over n501–MMAE. These results demonstrate the potential of fusing small‐sized ADCs to αHSA to improve therapeutic efficacy, thus providing a new insight for extending the half‐life of single‐domain antibody conjugates to open the therapeutic window of solid tumor.

## 2 RESULTS

### 2.1 Identification of n501–αHSA

**FIGURE 1:**
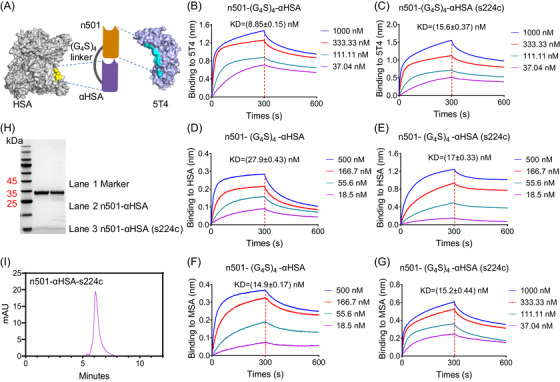
The design of n501–αHSA and its binding properties. (A) Structural illustration of n501–αHSA comprising n501 and αHSA linked via a linker (G_4_S)_4_. n501 and αHSA were colored in orange and purple, respectively. The HSA (PDB: 1AO6) was depicted as gray surface with binding epitopes highlighted in golden yellow and 5T4 (PDB: 4CNM) was depicted as light purple surface with binding epitopes highlighted in blue, respectively. (B–G) Binding kinetics of n501–(G_4_S)_4_–αHSA and n501–(G_4_S)_4_–αHSA (s224c) to 5T4, HSA, and MSA, as measured by BLI. (H) SDS‐PAGE analysis of n501–(G_4_S)_4_–αHSA and n501–(G_4_S)_4_–αHSA (s224c). (I) Size exclusion chromatography profile of n501–αHSA (s224c).

We fused n501 and αHSA with G_4_S linkers. According to previous reports, αHSA binds to the PSLAAD epitope of HSA,^27^ while n501 binds to eight leucine‐rich repeats of 5T4^12^ (Figure 1A). To explore the effects of the (G_4_S)_3_ and (G_4_S)_4_ linkers on antibody yield and affinity, we constructed n501–(G_4_S)_3_–αHSA, n501–(G_4_S)_4_–αHSA and their cysteine mutant variants n501–(G_4_S)_3_–αHSA (s219c), n501–(G_4_S)_4_–αHSA (s224c), respectively. The mutant variants are used to conjugate an agent‐linker conjugate mc‐vc‐PAB‐MMAE through cysteine. We summarized the yield and the binding ability to antigen 5T4, HSA, MSA of these two fusion protein forms and their cysteine mutant in Table S1. The yield of n501–(G_4_S)_4_–αHSA was 7.35 mg/L, higher than n501–(G_4_S)_3_–αHSA. The cysteine mutant variants had similar yields which were decreased compared with the nonmutant variants (Table S1).

We next used the biolayer interferometry (BLI) to measure the affinity of the cysteine variants to antigen 5T4. They showed similar *K*_D_ values except for n501–(G_4_S)_3_–αHSA (Figures 1B, C and S1B, C), which had reduced affinity (39.6 ± 0.54 nM). Since the cysteine mutation site of n501–αHSA variants is at position 84 of αHSA, and previous research showed that αHSA can also bind to MSA, we detected fusion protein binding to both HSA and MSA using enzyme‐linked immunosorbent assay (ELISA) and BLI. As shown in Figures S1D, E and Table S1, after cysteine mutation, the binding capacity of n501–(G_4_S)_3_–αHSA (s219c) to HSA and MSA decreased by 3.1 and 2.3 times, respectively (681.3 vs. 218.6 nM and 89.58 vs. 38.59 nM). The *K*_D_ value also showed that the affinity of n501‐(G_4_S)_3_‐αHSA with HSA and MSA after cysteine mutation was weakened (31.8 ± 0.57 vs. 26.2 ± 0.63 nM and 24.7 ± 0.58 vs. 12.6 ± 0.21 nM) (Figures S1F–I). However, the binding ability of n501–(G_4_S)_4_–αHSA to HSA or MSA changed only slightly before and after cysteine mutation (Figures S1D–E). ELISA results showed that after cysteine mutation, the binding capacity of n501–(G_4_S)_4_–αHSA (s219c) to HSA and MSA was slightly decreased by 1.4 and 1.3 times, respectively (119.2 vs. 82.76 nM and 40.68 vs. 30.41 nM) (Figures S1D and E). BLI assay did not show any change in *K*_D_ values for binding to HSA and MSA (17 ± 0.33 vs. 27.9 ± 0.43 nM and 15.2 ± 0.44 vs. 14.9 ± 0.17 nM) (Figures 1D–G). Based on these results, we selected n501–(G_4_S)_4_–αHSA (s224c) for follow‐up experiments and referred to it as n501–αHSA for simplicity. The purity and uniformity of n501–αHSA (s224c) were detected by sodium dodecyl sulfate polyacrylamide gel electrophoresis (SDS‐PAGE) and SEC‐HPLC (Figures 1H and I).

### 2.2 Conjugation with MMAE did not affect the binding specificity of n501–αHSA–MMAE

**FIGURE 2:**
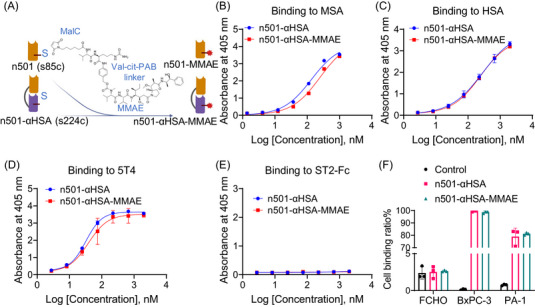
Binding specificity of n501–αHSA–MMAE to antigen of MSA, HSA, 5T4, and 5T4^+^ cells. (A) Schematic representation of the conjugation of n501–αHSA to the payload MMAE via the linker val‐cit‐PAB. (B–E) Binding capacity of the n501–αHSA and n501–αHSA–MMAE to the HSA, MSA, 5T4, and irrelated antigen ST2‐Fc, as measured by ELISA, *n* = 3. (F) Binding capacity of n501–αHSA and n501–αHSA–MMAE to the 5T4^+/−^ cell lines, as measured by flow cytometry, *n* = 3.

To prepare n501 and n501–αHSA–drug conjugate, we chose MMAE, a highly potent anticancer agent disrupting tubulin metabolism, as our cytotoxic payload. We used a derivative of MMAE (vcMMAE) containing a dipeptide Val‐Cit and a self‐immolative linker para‐aminobenzyloxycarbonyl (PAB) between the maleimide group (MC) and the cytotoxic drug (MMAE) groups which, after hydrolysis by cathepsin B in the lysosomes, releases the active drug form and causes the death of a cell^28^ (Figure 2A). The purity and stability of n501 and n501–αHSA were not affected by MMAE conjugation (Figure S1J). After analyzing the DAR values by reversed‐phase high performance liquid chromatography (RP‐HPLC), the results showed that n501–s85c and n501–αHSA–s224c are both site‐specific conjugation of MMAE to cysteine residues resulting in a DAR of 1 (Figures S2A and B). Since there is only one cysteine residue on each antibody, the theoretical DAR is 1. However, during the coupling process, some antibodies are not coupled to MMAE, causing them to appear in the form of dimer, so the average DAR is less than 1. Therefore, the average DAR of n501–MMAE and n501–αHSA–MMAE are 0.88 and 0.71 respectively (Figures S2A and B). ELISA results showed that the binding ability of n501‐ αHSA to MSA, HSA and 5T4 was not reduced after conjugating with vcMMAE (Figures 2B–D). Flow cytometry also demonstrated that the conjugation of MMAE did not affect the binding of n501–αHSA–MMAE to the 5T4^+^ cell line (Figures 2F and S3). n501–αHSA and n501–αHSA–MMAE did not show any binding to unrelated antigens and 5T4^−^ cell line FCHO (Figures 2E, F and S3).

### 2.3 n501–αHSA–MMAE had cytotoxicity against 5T4‐expressing cells

**FIGURE 3:**
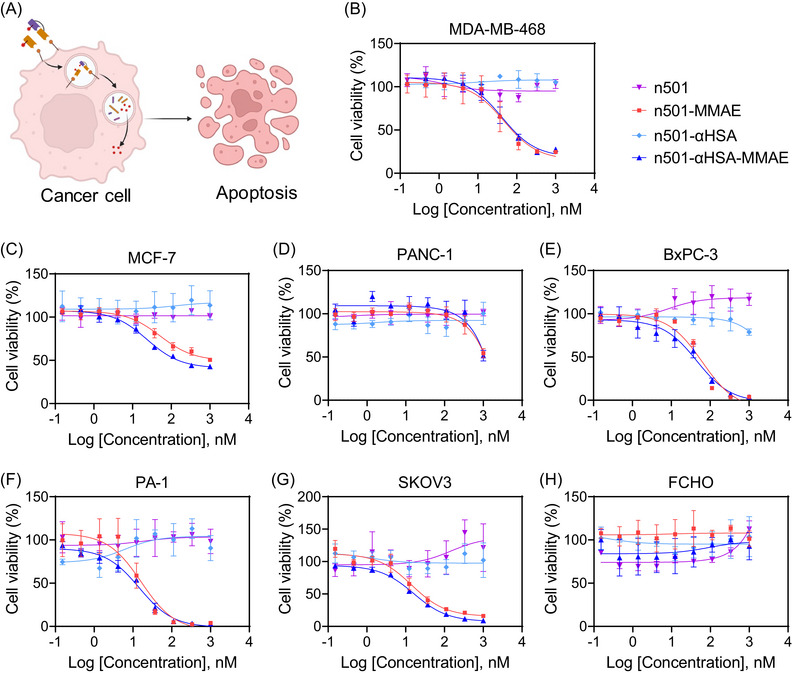
In vitro cell cytotoxicity of n501–αHSA–MMAE. (A) Schematic diagram of n501–MMAE and n501–αHSA–MMAE killing tumor cells. (B–H) Cell cytotoxicity of n501, n501–αHSA and corresponding drug conjugates in MDA‐MB‐468, MCF‐7, PANC‐1, BxPC‐3, PA‐1, SKOV3, and FCHO cell lines (*n* = 3, Naked antibody in MDA‐MB‐468, PA‐1, and FCHO, *n* = 2).

ADCs bind to tumor surface‐associated antigens and are internalized and undergo lysosomal degradation, releasing small molecule toxins that damage tumor cells (Figure 3A). We evaluated the in vitro cytotoxic activity of the conjugates against the high 5T4 expressing cell lines MDA‐MB‐468, MCF‐7, PANC‐1, medium 5T4 expressing cell line BxPC‐3, PA‐1, SKOV3 (Figure S4), and 5T4 negative cell line FCHO. Unconjugated antibodies were used as controls. As shown in Figures 3B–H and Table 1, n501–MMAE and n501–αHSA–MMAE showed comparable cytotoxicity against MDA‐MB‐468 (IC_50_ 46.71 vs. 41.82 nM) (Figure 3B), PA‐1 (IC_50_ 16.32 vs. 14.57 nM) (Figure 3F) and SKOV3 (IC_50_ 14.27 vs. 16.01 nM) (Figure 3G). Differently, n501–αHSA–MMAE had stronger cytotoxicity to BxPC‐3 and MCF‐7 than n501–MMAE (IC_50_ 45.43 vs. 61.98 nM, 3.5 vs. 51.22 nM) (Figures 3E and 3C). In addition, n501–MMAE and n501–αHSA–MMAE only killed PANC‐1 cells at a high concentration of 1000 nM (Figure 3D). No cytotoxicity was observed with n501–MMAE and n501–αHSA–MMAE against 5T4 negative FCHO cells (Figure 3H). These results indicated that n501–MMAE and n501–αHSA–MMAE specifically target and potently kill 5T4 expressing cells.

**TABLE 1:** In vitro cytotoxicity of anti‐5T4 ADC in different cancer cell lines.

Cell lines	Cancer type	Relative 5T4 density on cell	In vitro cytotoxicity (IC_50_ in nM)	
			n501–MMAE	n501–αHSA–MMAE
MDA‐MB‐468	Breast	+++	46.71	41.82
MCF‐7	Breast	+++	51.22	23.5
PANC‐1	Pancreatic	++	>1000	>1000
BxPC‐3	Pancreatic	+	61.98	45.43
PA‐1	Ovarian	+	16.32	14.57
SKOV3	Ovarian	+	14.27	16.01
FCHO	Noncancer cell	–	>1000	>1000

Abbreviation: IC_50_, half maximal inhibitory concentration.

### 2.4 αHSA effectively prolonged the serum half‐life of n501–αHSA–MMAE

**FIGURE 4:**
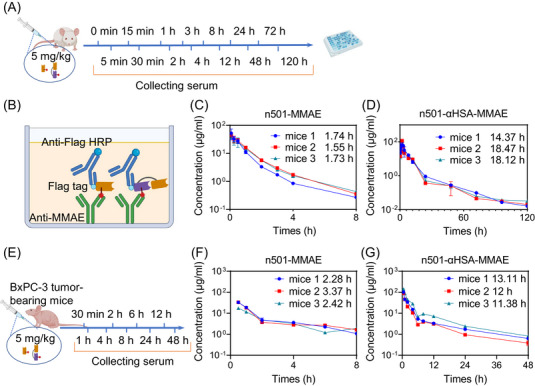
n501 fusion to αHSA extends serum half‐life. (A) Schematic diagram of n501–MMAE and n501–αHSA–MMAE biodistribution in healthy BALB/c mice. (B) Schematic diagram of n501–MMAE and n501–aHSA–MMAE in serum detected by ELISA. (C and D) PK of 5 mg/kg n501–MMAE and n501–αHSA–MMAE in healthy BALB/c mice (*n* = 3). (E) Schematic diagram of n501–MMAE and n501–αHSA–MMAE in BxPC‐3 tumor‐bearing mice. (F and G) PK of 5 mg/kg n501–MMAE and n501–αHSA–MMAE in BxPC‐3 tumor‐bearing mice (*n* = 3).

To verify the effect of prolonged half‐life of n501–αHSA–MMAE, we injected BALB/c mice and BxPC‐3 xenograft nude mice model with 5 mg/kg n501–MMAE and n501–αHSA–MMAE intravenously and collected plasma at different time points (Figures 4A and E), and tested the half‐life of the two ADCs in the mice. We used anti‐MMAE coating 96 well plates and detected the concentration of antibody‐conjugated‐MMAE in the plasma to exclude the interference of naked antibodies (Figure 4B). The results are shown in Figures 4C and D and Table 2, the half‐life of n501–MMAE in the mice was only 1.68 ± 0.11 h. The peak concentration *C*_max_ of n501–MMAE was 44.9 ± 7.4 µg/mL, while the half‐life of n501–αHSA–MMAE is 10 times longer than that of n501–MMAE (16.99 ± 1.86 h), and its *C*_max_ value was also higher than n501–MMAE (84.4 ± 23.16 µg/mL). In order to detect the extension of the half‐life of n501–αHSA–MMAE in tumor‐bearing mice, we used the BxPC‐3 xenograft mice model (Figure 4E). The results showed that in tumor‐bearing mice, the *C*_max_ of n501–MMAE and n501–αHSA–MMAE in the serum were all reduced, which were 27.89 ± 9.04 and 55.76 ± 14.56 µg/mL, respectively. The possible reason is that the accumulation of ADC in the tumor leads to a reduction in serum concentration. In addition, consistent with the results in wild‐type mice, in the BxPC‐3 xenograft mouse model, n501–αHSA–MMAE was also able to extend the half‐life nearly 5 times compared with n501–MMAE (12.2 ± 0.87 vs. 2.69 ± 0.59 h) (Figures 4F and G). These results indicate that αHSA fusion effectively extends the serum half‐life of n501–MMAE in healthy mice and the BxPC‐3 xenograft nude mice model.

**TABLE 2:** Pharmacokinetic parameters of ADC in BALB/c mice.

Group		*C*_max_ (µg/mL)	*T*_1/2_ (h)
Healthy BALB/c mice	n501–MMAE	44.9 ± 7.4	1.68 ± 0.11
	n501–αHSA–MMAE	84.4 ± 23.16	16.99 ± 1.86
BxPC‐3 xenograft nude mice	n501–MMAE	27.89 ± 9.04	2.69 ± 0.59
	n501–αHSA–MMAE	55.79 ± 14.56	12.2 ± 0.87

Abbreviations: *C*_max_, peak concentration; *T*_1/2_, plasma half‐life time.

### 2.5 αHSA effectively increased drug accumulation within the tumor

**FIGURE 5:**
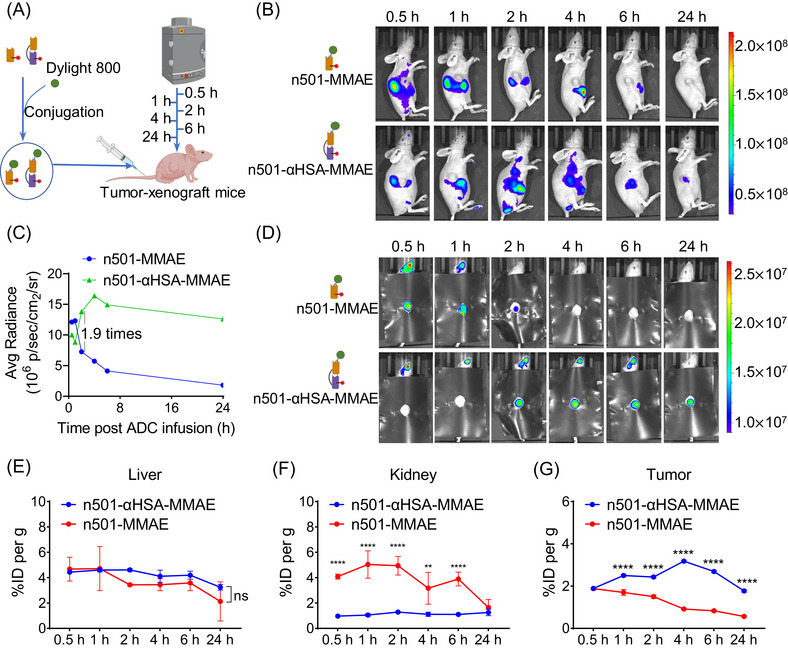
n501 fusion to αHSA extends serum half‐life and prolongs tumor accumulation. (A) Experimental design for fluorescence imaging of DyLight 800‐labeled n501–MMAE and n501–αHSA–MMAE biodistribution in BxPC‐3 xenografts. The whole bodies and tumor sites of mice were imagined at 0.5, 1, 2, 4, 6, and 24 h after treated with 5 mg/kg n501–MMAE and n501–αHSA–MMAE. (B) The whole body of mice were imaged at several time points post‐injection of DyLight800‐labeled n501–MMAE and n501–αHSA–MMAE using the caliper IVIS Lumina II system. (C and D) The tumor sites of mice were imaged after post‐injection of DyLight800‐labeled n501–MMAE and n501–αHSA–MMAE using the caliper IVIS Lumina II system. (C) The corresponding values of average fluorescence radiance (p/s/cm^2^/sr), as determined by Living Image software. (D) Imaging pictures of the tumor site after the body was covered with black tape. (E and F) BxPC‐3 xenograft mouse model was intravenously injected with 10 mg/kg of 501‐MMAE and n501–αHSA–MMAE at 0 h, 0.5, 1, 2, 4, 6, and 24 h. Calculating the percentage of injected dose per gram of tissue (%ID per g) in the liver (E), kidney (F) and tumor tissue (G) (*n* = 3).

In order to compare the distribution of n501–MMAE and n501–αHSA–MMAE in tumors, we used semi‐quantitative method NIR fluorescence imaging to monitor the distribution of ADCs in mice, we labeled these two ADCs with DyLight 800 (Figure 5A), a near‐infrared fluorescence that can be detected by an in vivo imager at 777 nm excitation light and 800 nm emission light. After the labeled ADCs were injected, the mice were imaged at different times (Figure 5A). As shown in Figure 5B, after n501–MMAE was injected, it would be quickly distributed to the kidneys and liver of mice. Due to the strong fluorescence signal of the organs, the antibody signal at the tumor site was almost invisible. The antibody signal was completely undetectable at 24 h. For n501–αHSA–MMAE, within 1 h after administration, the fluorescence signal of n501–αHSA–MMAE in the liver and kidney was less than n501–MMAE. After 2 h, n501–αHSA–MMAE began to gather at the tumor site, and can still be detected at the tumor site at 24 h. In order to exclude the fluorescent signals of the liver and kidney to detect the ADCs distribution at the tumor site, we covered the body of the mice with black tape, revealing only the tumor site. As shown in Figure 5D, the signal of n501–MMAE in tumor can only be detected at 0.5–2 h, and the signal of n501–αHSA–MMAE in the tumor site reached peak at 2 h, which is 1.9 times than n501–MMAE (Figure 5C). The result was consistent with the half‐life detection (Figure 4D). Surprisingly, the intratumoral concentration of n501–αHSA–MMAE remained high at 24 h (Figures 5C and D).

Since strong fluorescence in the liver and kidney in NIR fluorescence imaging, we tested samples from the liver, kidney, brain and tumor tissues. The results showed that the concentrations of n501–αHSA–MMAE and n501–MMAE in livers were comparable (Figure 5E), while the accumulation of n501–αHSA–MMAE in the kidneys was significantly lower than that of n501–MMAE (Figure 5F). The quantitative results for tumors were consistent with the NIR fluorescence imaging. The accumulation of n501–αHSA–MMAE in tumors was significantly higher than n501–MMAE group (Figure 5G). These results demonstrated that the fusion of αHSA to n501 effectively enhanced its accumulation at the tumor site.

### 2.6 n501–αHSA–MMAE showed potent antitumor effect and was more effective in PA‐1 xenograft tumor model

**FIGURE 6:**
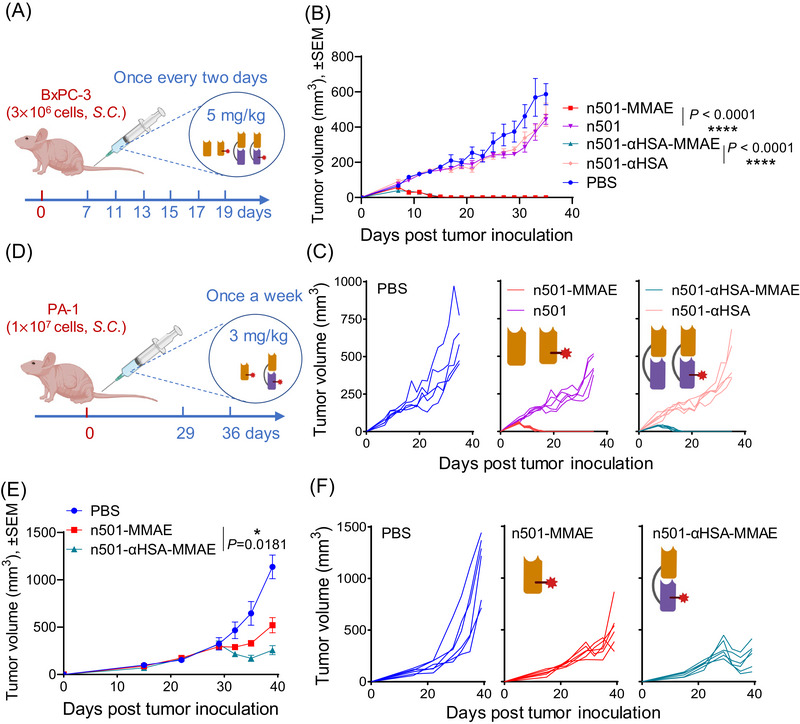
Therapeutic efficacy of n501–αHSA–MMAE in BxPC‐3 and PA‐1 xenograft mouse models. (A) Schematic representation of in vivo experiments in BxPC‐3 tumor‐bearing mouse model. (B and C) BxPC‐3 xenografted mice were treated with 5 mg/kg n501–MMAE and n501–αHSA–MMAE intravenously for 6 times every 2 days with PBS, with n501 and n501–αHSA as vehicle control. Data are expressed as mean ± SEM, *n* = 5. The average tumor volume (mm^3^) (B) and individual tumor volume (C) were measured. (D) Schematic representation of in vivo experiments in PA‐1 tumor‐bearing mouse model. (E and F) PA‐1 xenografted mice were treated intravenously one dose weekly with 3 mg/kg n501–MMAE and n501–αHSA–MMAE, with PBS as vehicle control. Data were expressed as mean ± SEM, *n* = 6. The average tumor volume (mm^3^) (E) and individual tumor volume (F) were measured.

In order to verify the antitumor effect of n501–αHSA–MMAE after the half‐life is extended, we examined its antitumor effect in the BxPC‐3 and PA‐1 xenograft tumor models. The results are shown in Figures 6 and S5. BxPC‐3 tumor‐bearing mice were administered 5 mg/kg of n501–MMAE and n501–αHSA–MMAE six doses every other day (Figure 6A). Naked antibodies were used as controls. Tumor growth was significantly suppressed after the first injection of both ADCs, and the tumor size gradually became smaller until undetectable, and it did not relapse in the later stage (Figures 6B and C). Mouse weight was unaffected by either ADC or naked antibody at 5 mg/kg (Figure S5B). After 35 days post tumor inoculation, mice were executed by cervical dislocation and their tumors were weighed—the mice in the n501–MMAE and n501–αHSA–MMAE treatment groups were completely healed, and the naked antibody had no effect on the tumor weight of the tumor‐bearing mice (Figure S5C).

To compare therapeutic effects of half‐life extension, we treated BxPC‐3 tumor‐bearing mice with 3 mg/kg of n501–MMAE and n501–αHSA–MMAE once a week or twice a week (Figures S6A and S6D). Both ADCs inhibited the tumor growth at 3 mg/kg, though no significant difference was seen between the two groups (Figures S6B, C and S6E, F).

Then, we selected human ovarian teratoma cell PA‐1, a cell line that was more sensitive to n501–MMAE and n501–αHSA–MMAE in vitro, to construct a tumor‐bearing mice model. When the tumor grows to 300−400 mm^3^, mice were injected weekly with n501–MMAE and n501–αHSA–MMAE (Figure 6D). Tumor volume of the mice in the n501–αHSA–MMAE treatment group was significantly smaller than that of the n501–MMAE group (Figures 6E and F). After the mice were executed on the 39th day of tumor‐bearing, the tumors were weighed and photographed. Tumor size and weight of the mice in the n501–αHSA–MMAE group were significantly lower than that of the n501–MMAE group (Figures S5D–F). These results demonstrated that extending half‐life effectively improves the antitumor efficacy of n501–αHSA–MMAE in PA‐1 xenograft tumor model.

### 2.7 n501–αHSA–MMAE exhibited comparable toxicity to n501–MMAE at high doses

**FIGURE 7:**
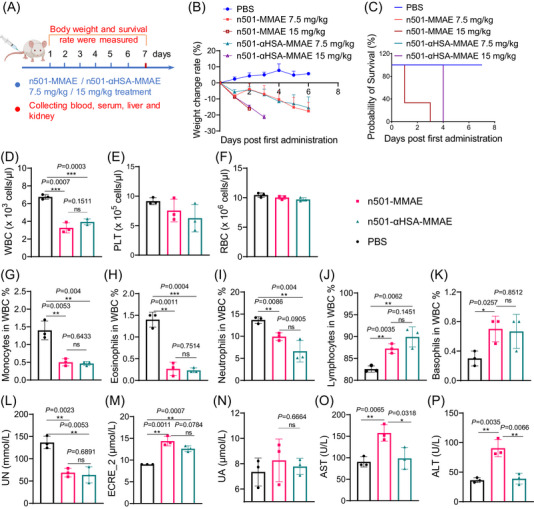
Safety evaluation of n501–αHSA–MMAE in BALB/c mice. (A) Schematic of treatment regimen. Mice were treated with 7.5 mg/kg or 15 mg/kg drugs daily for six doses. (B) The body weight change rate of mice was measured (*n* = 3). (C) The survival rate of mice was measured (*n* = 3). (D–K) Blood was collected after administration of 7.5 mg/kg for six doses. The amount of white blood cells (WBC) (D), platelet cells (PLT) (E), and red blood cells (RBC) (F) were detected, and the ratio of monocytes (G), eosinophils (H), neutrophils (I), lymphocytes (J), basophils (K) in WBC were measured. (L–P) The serum was collected to test UN (L), ECRE_2 (M), UA (N), AST (O), and ALT (P) (*n* = 3).

In vivo imaging showed that n501–αHSA–MMAE had less kidney accumulation than n501–MMAE (Figure 5B). Therefore, we explored whether n501–αHSA–MMAE is safer than n501–MMAE. In the previous part of the results, 5 mg/kg doses of both ADCs did not affect mouse weight, so we increased the injection dose and frequency to explore the safety of n501–αHSA–MMAE. Mice were injected with 7.5 or 15 mg/kg of n501–MMAE and n501–αHSA–MMAE for 6 days (Figure 7A). As shown in Figures 7B, C and S7A, 15 mg/kg n501–MMAE and n501–αHSA–MMAE caused serious weight loss in mice, and mice died on the third day after two doses of 15 mg/kg of n501–MMAE. Mice in 15 mg/kg n501–αHSA–MMAE group all died after the third dose at day 4, indicating stronger side effects at this high dose. 7.5 mg/kg of n501–MMAE and n501–αHSA–MMAE also caused weight loss in mice, but did not cause death in mice after six doses.

Since MMAE‐related drugs can cause disorders of the blood system, we tested the cell changes in the blood of mice after injecting 7.5 mg/kg of n501–MMAE and n501–αHSA–MMAE. The results showed that after mice were injected with n501–MMAE and n501–αHSA–MMAE, the number of white blood cells in the blood were decreased (Figure 7D). Specifically, the proportion of monocytes, eosinophils, and neutrophils decreased (Figures 7G–I), and the proportion of lymphocytes and basophils increased (Figures 7J and K), but there was no significant difference between the two groups of n501–MMAE and n501–αHSA–MMAE. In addition, the injection of n501–MMAE and n501–αHSA–MMAE did not affect the number of platelets and red blood cells in the blood of mice (Figures 7E and F).

As the accumulation of two ADCs in the liver and kidney was previously observed, the serum of mice was collected to detect the biochemical indicators of the liver and kidney. The result showed that n501–MMAE and n501–αHSA–MMAE significantly reduced the level of uric nitrogen (UN) and increased the level of enzymatic creatinine‐2 (ECRE_2) (Figures 7L and M). There was no significant effect on uric acid (UA) (Figure 7N), and there was no significant difference between the two groups of n501–MMAE and n501–αHSA–MMAE. It was found that the aspartate aminotransferase (AST) and alanine aminotransferase (ALT) levels of mice in the n501–MMAE group increased significantly (Figures 7O and P), which was markedly different from the n501–αHSA–MMAE group. H&E staining of liver and kidney tissues showed that n501–MMAE and n501–αHSA–MMAE did not cause inflammatory cell infiltration and necrosis (Figure S7B). The above results showed that n501–MMAE and n501–αHSA–MMAE could produce certain toxic effects at high doses, but n501–αHSA–MMAE has better liver safety than n501–MMAE.

## 3 DISCUSSION

The dense structure of tumor tissue presents a barrier for penetration and distribution of traditional IgG drugs, making it difficult to reach tumor cells distant from vasculature and resulting in poor therapeutic efficacy.^5^ To address this limitation, researchers have focused on smaller antibody fragments such as Fab, scFv, and VH.^29^, ^30^, ^31^ While these fragments may improve tumor penetration, their rapid clearance due to shorter half‐lives compared with full‐size antibodies requires frequent administration, greatly restricting clinical utility.

Previous studies by our group and others have explored the diagnostic and therapeutic potential of single‐domain antibodies for various diseases.^12^, ^32^, ^33^, ^34^, ^35^ The small size of single‐domain antibodies results in rapid renal clearance. This property is ideal for diagnostic imaging purposes, such as conjugated to a multitude of radioisotopes for PET and SPECT imaging, as well as second near infrared bioimaging of metastatic tumors.^33^, ^36^ However, for effective tumor therapy, longer circulation half‐life and retention time may be advantageous as it may allow for a gradual accumulation within tumors. Therefore, methodologies to extend circulation half‐life of single‐domain antibodies without substantially increasing size are in demand.

At present, methods to extend the half‐life of nanobodies mainly include Fc fusion, PEG modification, and fusion with albumin or αHSA. Among them, the fusion technology of nanobodies and Fc utilizes the combination of Fc and FcRn to extend the half‐life. The binding of the Fc region of IgG to FcRn is pH dependent. It binds FcRn with high affinity under intracellular acidic conditions and rapidly dissociates from FcRn under blood conditions, thereby extending the half‐life through this receptor‐mediated recycling mechanism.^37^, ^38^ However, this method inevitably increases the molecular size of the nanobody, thereby reducing its advantage of stronger tumor penetration. In addition, PEG modification is also a common method to extend the half‐life. PEGylation modification will change the physical and chemical properties of the drug, including conformation, electrostatic binding, hydrophobicity, and so on. These physical and chemical changes increase the retention time of the drug in the body, increase the plasma half‐life, prolong the absorption time.^39^ However, in recent years, studies have found that PEG injection is immunogenic and induces the body to produce anti‐PEG antibodies, which affects the half‐life and causes hypersensitivity reactions.^40^ To extend the half‐life of n501–MMAE while maintaining its antitumor effects, we fused n501 with a nanobody that can bind to HSA. αHSA exploits FcRn‐mediated recycling to prolong PK of fused proteins with minimal increase in size (∼12 kDa).^41^, ^42^ HSA can extravasate from the bloodstream to reach the lymphatic system and accumulate in cancerous areas, thus providing not only a mechanism for prolonging the half‐life of therapeutic proteins but also additional potential advantages for cancer treatment.^43^, ^44^ The fusion protein n501–αHSA and its cysteine variants could bind 5T4 with cross‐reactivity towards serum albumin from different species (Figures 1B–G), including human and mice. This enabled efficacy and toxicology evaluation in mouse models without the need to develop species‐specific surrogates.

Specific targeted in vitro cytotoxicity of n501–αHSA–MMAE was quantified in comparison with a relevant ADC n501–MMAE. n501–αHSA–MMAE demonstrated cytotoxicity in 5T4^+^ cell lines (Figure 3). In addition, n501–MMAE and n501–αHSA–MMAE showed comparable cytotoxicity in vitro, which is attributed to equivalent MMAE drug content in the two conjugates.

Albumin accumulates in inflamed and malignant tissues and is usually considered to involve an effect called enhanced permeability and retention, which occurs due to extensive neovascularization in these tissues and the combined effects of leaking capillary networks and damaged lymphatic drainage.^45^, ^46^ Albumin and other plasma proteins are also catabolized by tumors, providing nutrition and energy for fast‐growing tumors.^47^, ^48^ n501–αHSA–MMAE can bind albumin and possibly accumulate in tumor with the help of albumin. Compared with n501–MMAE, n501–αHSA–MMAE demonstrated enhanced tumor accumulation and maintained a longer time infiltrating the tumor (Figure 5).

The PK properties of ADCs depend on the binding energy, mAb, and cytotoxic payload, but are mainly affected by the mAb.^49^ There are currently 14 ADCs approved for the treatment of tumors, all of which are in IgG format, and most of them are IgG1. This structure has a long half‐life due to its Fc structure, ranging from a few to more than 10 days.^50^ ADCs with high DAR values have been shown to have higher clearance than their unconjugated mAb or those with lower DAR.^51^, ^52^ In our previous study, we compared the difference in half‐life of IgG format ADC and sdADC targeting 5T4. The half‐life of IgG format ADC was 57.2 h in wild‐type mice, which was 37‐fold longer than that of sdADC.^12^ Our results showed that the half‐life extended over 10‐fold in wild‐type mice and fivefold prolonged serum half‐life in BxPC‐3 xenograft tumor models with only a twofold increase in molecular weight to 27 kDa (Figure 4).

To compare the safety of the n501–αHSA–MMAE and n501–MMAE, we used high doses and injected for 6 days to distinguish the differences between the two conjugates. But surprisingly, n501–αHSA–MMAE and n501–MMAE have similar toxicity to blood, liver and kidneys, except that the n501–αHSA–MMAE group does not have elevated ALT and AST. This toxicity is similar to the clinical toxicity of MMAE conjugated ADCs with neutropenia and liver toxicity.^53^ In addition, during the quantification of renal accumulation in mice, we were pleasantly surprised to find that the renal accumulation level in the n501–αHSA–MMAE group was significantly lower than that in the n501–MMAE group, which may be due to the fact that the glomerular gap is about 8 nm, will filter out proteins smaller than 25 kDa, while fusion with αHSA increases their molecular weight to ∼27 kDa. Therefore, it is not cleared by the kidneys thereby increasing half‐life and safety.^54^

To further evaluate the therapeutic potential of n501–αHSA–MMAE, we also examined its antitumor activity in BxPC‐3 and PA‐1 xenograft mouse models. As expected, n501–αHSA–MMAE exhibited antitumor efficacy (Figures 6 and S5). Owing to its extended half‐life, dosing frequency was reduced to twice weekly or weekly, compared with every other day for n501–MMAE. In the BxPC‐3 xenograft mouse model, n501–αHSA–MMAE did not show improved antitumor effects over n501–MMAE (Figure S6), possibly because it contained half the MMAE drug content by dose. Interestingly, n501–αHSA–MMAE achieved the same therapeutic efficacy compared with n501–MMAE, likely due to the extended half‐life of n501–αHSA and the increased tumor accumulation mediated by albumin binding. In the PA‐1 xenograft mouse model of large tumors with relatively rapid growth, n501–αHSA–MMAE demonstrated superior antitumor activity compared with n501–MMAE (Figures 6D–F and S5D–F). The fusion of αHSA not only reduces the hepatotoxicity of n501–MMAE, but also results in less renal accumulation and a longer half‐life, which results in unnecessary frequent drug injections to achieve tumor inhibition effects and is more conducive to clinical application. However, although the fusion of αHSA has a very small increase in the molecular weight of single domain antibodies compared with Fc fusion proteins, it still inevitably doubles the molecular weight of sdADC. It is unknown whether the increased molecular weight affects the tumor penetration of n501–αHSA–MMAE. In order to solve this problem, we will subsequently design a linker between n501 and αHSA that can be cleaved in the tumor microenvironment, so that its tumor penetration ability remains unchanged and achieve better antitumor effects in tumors.

In conclusion, we genetically fused the αHSA nanobody to the fully human single‐domain antibody n501 to generation the fusion protein n501–αHSA and its corresponding ADC n501–αHSA–MMAE. Compared with n501–MMAE, n501–αHSA–MMAE maintained binding abilities and in vitro cytotoxicity to 5T4^+^ tumor cells. Moreover, n501–αHSA–MMAE exhibited a dramatically extended half‐life and prolonged exposure in mice with the t_1/2_ nearly 10‐fold higher than that of n501–MMAE. n501–αHSA–MMAE also showed enhanced tumor accumulation and retention attributed to its prolonged exposure. Notably, n501–αHSA–MMAE showed enhanced antitumor efficacy over n501–MMAE in PA‐1 xenograft mouse models. These features make fusion to αHSA an attractive approach to improve the therapeutic potential of small‐sized ADCs or PDCs for cancer treatment.

## 4 MATERIALS AND METHODS

### 4.1 Cell lines

All tumor cell lines were of human origin. Pancreatic carcinoma cell PANC‐1 was obtained from the Fudan Institutes of Biomedical Sciences Cell Center (Shanghai, China). Pancreatic carcinoma cell BxPC‐3, ovarian carcinoma cell PA‐1, breast carcinoma cell MDA‐MB‐468, hepatocellular carcinoma cell Huh‐7, and Chinese hamster ovary cell FCHO were purchased from Cell Bank of the Chinese Academy of Sciences (Shanghai, China). BxPC‐3 were maintained in RPMI 1640 medium (Hyclone) supplemented with 10% fetal bovine serum (FBS; Yeasen), 100 U/mL penicillin (Gibco), and 100 µg/mL streptomycin (Gibco). Other cell lines were maintained in DMEM medium (Hyclone) with 10% FBS (Gibco), 100 U/mL penicillin (Gibco), and 100 µg/mL streptomycin (Gibco). All cells were maintained in a humidified incubator at 37°C with 5% CO_2_.

### 4.2 Animals

All animal experiments were approved by the Experimental Animal Ethics Committee of Basic Medical Sciences at Fudan University. The approval Number is 20230428‐002. All BALB/c wild‐type mice and BALB/c nude mice were ordered from Shanghai Jikai Inc. and were fed on a free diet and water under specific pathogen‐free rooms.

### 4.3 Expression and purification of antibodies

The human single‐domain antibody n501 and n501–s85c were cloned into the pComb3x vector as previously described.^12^ The amino acid sequence of the anti‐HSA nanobody αHSA was obtained from previously report.^27^ The αHSA was fused into C‐terminal of n501 with (G_4_S)_3_ or (G_4_S)_4_ linker. The cDNA sequences encoding αHSA were reverse translated and synthesized by BGI genomics (Beijing, China) and codon optimization is suitable for *Escherichia coli* expression. The sequences of n501–αHSA were cloned into pComb3x vector. The N‐terminal of this vector contains the OmpA signal peptide (MKKTAIAIAVALAGFATVAQA) and hexahistidine (His6) at the C terminus for protein purification and Flag tag (DYKDDDDK). The n501–αHSA–s224c variant was constructed according to the instructions of the site‐directed mutagenesis kit (Yeasen, Shanghai).

The expression of n501, n501–αHSA, and their variants was *E. coli* HB2151. HB2151 was cultured at 37°C and 250 rpm/min and IPTG was added when OD_600_ reached 0.6 (about 2 h), then continued culturing at 30°C for 14 h. The *E. coli* cells were harvested by centrifugation and resuspended in buffer A [phosphate‐buffered saline solution (PBS) + 0.5 M NaCl] and lysed at 4°C by high pressure homogenizer at 10,000 psi for two to three times. The supernatant was collected after centrifugation at 8801 g for 30 min. After filtration with a 0.8 µm membrane, it was loaded over Ni‐NTA resin (GE Healthcare). The Ni‐NTA was washed with buffer (buffer A + 25 mM imidazole) and proteins were eluted in elution buffer (buffer A + 250 mM imidazole). For cysteine‐variants, 1 mM tris(2‐carboxyethyl) phosphine hydrochloride (TCEP) (Sigma–Aldrich) were added to the washing buffer and elution buffer to keep free cysteine. The collected fractions were concentrated using a 3 kDa Amicon ultracentrifugal concentrator (Millipore) and buffer exchanged into PBS for storage. The antibody concentration was detected by NanoDrop 2000 spectrophotometer (Thermo Fisher).

### 4.4 n501–αHSA purity determination

SDS‐PAGE was used to detected the size of n501–αHSA and high‐performance liquid chromatography (SEC‐HPLC) was used to determine the uniformity of n501–αHSA. For SDS‐PAGE, SmartPAGE™ 4−12% Bis‐Tris Protein precast gels (Smart Life‐Science, China) were used for SDS‐PAGE analysis, and approximately 5 µg of antibodies were loaded per well. After electrophoresis at 160 V for 40 min, the gel was stained with Coomassie Blue Fast Staining buffer (Beyotime Biotechnology, China) and destained with running water.

For SEC‐HPLC analysis, n501–αHSA was diluted to 1 mg/mL and 10 µL was added onto an Agilent Bio SEC‐3 column (150 Å, 3 µm, 4.6 × 150 mm) at 0.5 mL/min. PBS buffer was used as the mobile phase. The absorption peak at 280 nm was obtained to detect the uniformity of the antibody.

### 4.5 Preparation of n501–MMAE and n501–αHSA–MMAE

n501–s85c and n501–αHSA–s224c were mixed with threefold TCEP, and then 1 mM diethylenetriaminepentaacetic acid was added. The mixture was incubated at 25°C for 2 h with gently shaking. The reaction system was then slowly added dropwise to fivefold excess of vc‐MMAE and further incubated for 1 h at 25°C. Then, 10‐fold excess of cysteine was added over the drug linker to quench the reaction. The conjugates were added to a 3 kDa dialysis bag to dialyze in 2 L PBS for more than 24 h and replaced with fresh PBS after 12 h. Finally, n501–MMAE and n501–αHSA–MMAE were filtered by a 0.2‐µm filter under sterile conditions and stored at −80°C for further analysis.

### 4.6 Reversed‐phase high performance liquid chromatography

The average DAR value was detected using a PLRP‐S 1000A 2.1 × 150 mm, 8 µm column. The specific method was as follows: use 0.1% TFA–H_2_O as mobile phase A and 0.1% TFA–acetonitrile as mobile phase B. At a column temperature of 70°C, the sample concentration was adjusted to 1 mg/mL and was run at a flow rate of 0.5 mL/min for 20 min. At 0–3 min, phase A and phase B eluted at 70 and 30%, respectively. At 3−20 min, phase A and phase B eluted at a ratio of 1:1, and the absorption peak at the wavelength of 280 nm was collected.

### 4.7 Enzyme‐linked immunosorbent assay

Coating with 100 ng HSA, MSA, 5T4‐Fc, or ST2‐Fc in costar 96 half‐well plate (Corning #3690) at 4°C overnight. After washing, the plate was blocked for 1 h at 37°C with 5% BSA in PBS. Threefold serial dilution of antibodies were added and incubated at 37°C for 90 min. The wells were washed with PBST (PBS + 0.05% Tween 20) and were incubated with an anti‐Flag‐HRP at 37°C for 45 min (Sigma–Aldrich). The substrate ABTS was added to detect the enzyme activity. The optical density was read at 450 nm using a microplate reader (Biotek, Hercules, CA, USA).

### 4.8 Biolayer interferometry

The binding kinetics of n501–αHSA and n501 to 5T4 and HSA/MSA was measured using the Octet‐RED96 (ForteBio). Briefly, the AR2G biosensors were activated by reaction with 20 mM EDC (1‐ethyl‐3‐[3‐dimethylaminopropyl] carbodiimide hydrochloride) and 10 mM sulfo‐NHS (N‐hydroxysulfosuccinimide). 30 µg/mL of 5T4 and HSA/MSA were loaded on the AR2G biosensor and quenched with 1 M ethanolamine pH 8.5. After baseline with PBST buffer (PBS + 0.2% tween 20), the sensors were immersed with threefold serially diluted antibodies starting at 500 or 1000 nM in PBST for 300 s to associate, and then immersed into PBST for another 300 s to dissociate at 37°C. All the curves were fitted by 1:1 binding model using the Data Analysis software 10.0. *K*_D_ values were determined with *R* ^2^ values of greater than 95% confidence level.

### 4.9 Flow cytometry

The n501–αHSA and n501–αHSA–MMAE were labeled with EZ‐Link Sulfo‐NHS‐LC‐Biotin (Thermo Fisher Scientific) according to the instructions. The 5T4^+^ cell line BxPC‐3, PA‐1, and 5T4^−^ cell line FCHO were added into a 15 mL centrifuge tube at a density of 10^6^ cells and incubated with 1 µM labeled n501–αHSA and n501–αHSA–MMAE for 1.5 h. After washing twice with PBSF (PBS added with 0.5% FBS) and APC Streptavidin (Biolegend) was added for 30 min. After washing, the cells were detected by flow cytometry, and subsequent data were analyzed by Flow jo V10.

### 4.10 Evaluation the cytotoxicity of n501–αHSA–MMAE in vitro

Cytotoxicity assays performed in the presence of n501–MMAE and n501–αHSA–MMAE and their antibody control was assessed using cells plated at 5000 cells per well in medium with in flat bottom 96‐well cell culture plates (NEST Biotechnology, China). After 12 h, tumor cells were added with serial dilutions of n501–αHSA–MMAE or n501–MMAE in triplicate wells and incubated for 72 h at 37°C in a humidified 5% CO_2_ atmosphere. After mixing CCK8 reagent and complete medium at a ratio of 1:9, the cell culture medium was removed and CCK8 working solution was added for another 1–3 h. The absorbance was measured by a microplate reader at 450 nm, and the cell viability ratio (%) was calculated using the following formula: (A sample − A blank)/(A control − A blank) × 100%.

### 4.11 PK of n501–MMAE and n501–αHSA–MMAE in wild‐type mice and BxPC‐3 xenograft mice model

The PK of n501–αHSA–MMAE was evaluated in 8–10‐week‐old female wild‐type BALB/c mice and BxPC‐3 xenograft nude mice model. The mice were treated in replicas with 5 mg/kg of n501–MMAE and n501–αHSA–MMAE via tail vein injection, and blood was collected at different time points (0 min, 5 min, 15 min, 30 min, 1 h, 2 h, 3 h, 4 h, 8 h, 12 h, 1 day, 2 days, 3 days, and 5 days). For BxPC‐3 xenograft mice model, blood was collected at 0.5 h, 1 h, 2 h, 4 h, 6 h, 8 h, 12 h, 1 day, and 2 days. Serum concentrations of n501–MMAE and n501–αHSA–MMAE were detected by ELISA. Briefly, 96‐half‐well plates were coated with 100 ng/well of anti‐MMAE antibody at 4°C overnight. The plates were blocked with PBS containing 5% BSA and incubated with diluted serum or n501–MMAE and n501–αHSA–MMAE as standards. Serum samples of mice were 50‐fold diluted by PBS containing 1% BSA buffer. After incubation at 37°C for 90 min, the n501–αHSA–MMAE and n501–MMAE concentrations were detected by HRP‐anti‐Flag mAb (Sigma). The serum half‐life in mice was analyzed using Pharmacokinetics and Metabolism software.

### 4.12 Distribution of n501–αHSA–MMAE in a BxPC‐3 xenograft mouse model

n501–MMAE and n501–αHSA–MMAE were labeled by using DyLight 800 labeling kit. According to the instruction manual, briefly, 0.5 mL of n501–MMAE and n501–αHSA–MMAE were prepared in PBS. Borate buffer (40 µL) (0.67 M) was added to 0.5 mL of n501–MMAE and n501–αHSA–MMAE. The prepared protein was added into DyLight reagent, vortex gently to mix evenly, and incubated at room temperature in the dark for 1 h. Then, the purification resin provided in the kit was used to remove free dye.

Six‐weeks‐old female nude mice were subcutaneously inoculated with 5 × 10^6^ BxPC‐3 cells on right back. After 3−4 weeks, the tumor volume reached 200−300 mm^3^, the tumor‐bearing mice were imaged using the IVIS Lumina system (Caliper Life Science) after intravenous injection of 5 mg/kg DyLight 800 labeled n501–MMAE and n501–αHSA–MMAE, and imaging at different times (0.5, 1, 2, 4, 6 h, and 1 day). For distribution of ADCs in BxPC‐3 tumor site, mice were covered with black tape to block signals from liver and kidney. Data were analyzed by the Living Image software (Caliper LS). Regions of interest (ROIs) were drawn around the tumor areas, and the values of average fluorescence radiance (p/s/cm^2^/sr) from these ROIs were used to calculate tumor accumulation of the ADCs.

BxPC‐3 xenograft mice model was injected with 10 mg/kg of n501–MMAE and n501–αHSA–MMAE into the tail vein at 0, 0.5, 1, 2, 4, 6, and 24 h, respectively. Mice underwent cardiac perfusion to remove drug interference from the blood. The liver, kidney, brain, and tumor were taken and 150 µL RIPA lysis buffer for 40 mg of tissue was added. The tissue was grinded into homogenate and centrifuged at 13523 g for 10 min. The supernatant was taken for ELISA detection and quantification and the percentage of injected dose per gram of tissue (%ID per g) was calculated.

### 4.13 Human tumor xenograft mouse model

Tumor cells were suspended with 4 mg/mL Matrigel. According to the concentration of Matrigel provided in the instruction manual, Matrigel was adjusted to a concentration of 8 mg/mL with precooled PBS, and then it was mixed with BxPC‐3 and PA‐1 tumor cells in a ratio of 1:1 and were placed on ice. Six‐ to eight‐week‐old female nude mice were subcutaneously inoculated with 5 × 10^6^ or 3 × 10^6^ BxPC‐3 cells; for PA‐1 tumor model, mice were subcutaneously inoculated with 1 × 10^7^ cells. When the tumor volumes reached 50−100 mm^3^, mice were randomly divided into several groups (*n* = 5/6 per group). The mice were treated intravenously with PBS, 5 mg/kg n501, n501–MMAE, n501–αHSA, and n501–αHSA–MMAE every other day for a total of six doses. To compare the efficacy of n501–MMAE and n501–αHSA–MMAE, PA‐1 xenograft models were treated with 3 mg/kg n501–MMAE and n501–αHSA–MMAE once a week. BxPC‐3 xenograft models were treated with n501–MMAE and n501–αHSA–MMAE ADCs twice a week or once a week. Tumor volumes were measured using electronic calipers and calculated using the following formula: Volume (mm^3^) = *L* × *W* × *W*/2, where *L* and *W* represent the largest and smallest tumor diameters, respectively.

### 4.14 Safety of n501–αHSA–MMAE in healthy mice

Healthy female 18–21 g BALB/c mice were administered PBS, n501–MMAE, and n501–αHSA–MMAE (7.5 mg/kg and 15 mg/kg) once per day for 6 days. Blood was collected for hematology and serum chemistry analysis. The serum was used to detect AST, ALT, UN, ECRE‐2, and UA. Liver and kidney tissues from each mouse were also collected, fixed with 4% formalin, and stained with hematoxylin and eosin staining.

### 4.15 Statistical analysis

Statistical analyses were performed using GraphPad Prism Software version 8.0. (San Diego, CA, USA). Data were compared using two‐tailed unpaired Student's *t*‐test, and *p* < 0.05 was considered statistically significant, with different levels denoted as *p < 0.05, **p < 0.01, and ***p < 0.001, respectively.

## AUTHOR CONTRIBUTIONS

Y. W. and T. Y. initiated, planned, and supervised the project. Q. L. performed most of the experiments with assistance from Q. L., Y. K., Y. Z., A. H., Q. L., and Y. K. collected data. Q. L. conceived and wrote the paper. Y. W. and T. Y. revised and edited the final version of the manuscript. All authors have read and approved the final manuscript.

## CONFLICT OF INTEREST STATEMENT

The authors declare there are no conflicts of interest.

## ETHICS STATEMENT

All animal experiments were conducted according to the guidelines of the National Health and Medical Research Council (NHMRC). The approval Number is 20230428‐002.

## Supporting information

Supporting Information

Supporting Information

## Data Availability

The data that support the findings of this study are available from the corresponding author upon reasonable request.
