# Supplementary material for: Half‐life extension of single‐domain antibody–drug conjugates by albumin binding moiety enhances antitumor efficacy
Source: MedComm (2020). 2024 May 9;5(5):e557. doi: 10.1002/mco2.557 (PMC11082534; doi:10.1002/mco2.557)
Supplement: Supplementary file 1 — Supporting Information [file MCO2-5-e557-s002.docx]

Table S1. Effects of different linkers on antibody yield and affinity

| **Antibody** | **Yield (mg / L)** | **EC50 (nM, binding to HSA)** | **EC50 (nM, binding to MSA)** | **KD (nM,**  **binding to 5T4)** | **KD (nM,**  **binding to HSA)** | **KD (nM,**  **binding to MSA)** |
| --- | --- | --- | --- | --- | --- | --- |
| **n501- (G_4_S)_3_ -αHSA** | 6.26 | 218.6 | 38.59 | 13.2±0.38 | 26.2±0.63 | 12.6±0.21 |
| **n501- (G_4_S)_3_ -αHSA (s219c)** | 4.23 | 681.3 | 89.58 | 39.6±0.54 | 31.8±0.57 | 24.7±0.58 |
| **n501- (G_4_S)_4_ -αHSA** | 7.35 | 82.76 | 30.41 | 8.85±0.15 | 27.9±0.43 | 14.9±0.17 |
| **n501- (G_4_S)_4_ -αHSA (s224c)** | 4.07 | 119.2 | 40.68 | 15.6±0.37 | 17±0.33 | 15.2±0.44 |
