# Supplementary material for: Half‐life extension of single‐domain antibody–drug conjugates by albumin binding moiety enhances antitumor efficacy
Source: MedComm (2020). 2024 May 9;5(5):e557. doi: 10.1002/mco2.557 (PMC11082534; doi:10.1002/mco2.557)
Supplement: Supplementary file 2 — Supporting Information [file MCO2-5-e557-s001.pdf]

Figure S1

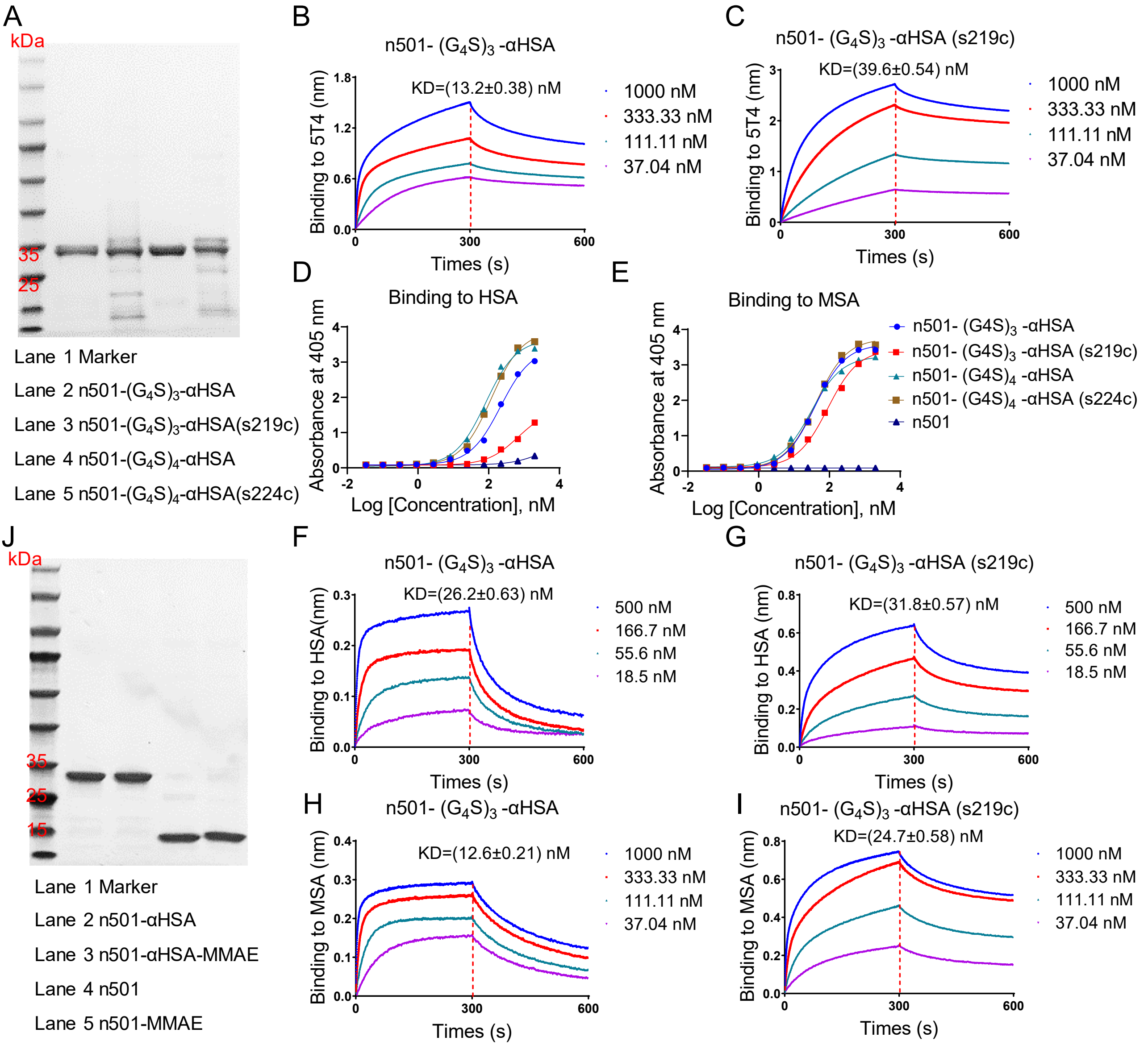

Figure S1. The design of n501-αHSA and its binding ability.

(A) SDS-PAGE analysis of n501-$(G_4S)_3$-αHSA, n501-$(G_4S)_4$-αHSA and their cysteine mutation variants. (B-C, F-G) Binding kinetics of n501-$(G_4S)_3$-αHSA and n501-$(G_4S)_3$-αHSA (s219c) to 5T4, HSA and MSA, respectively, as measured by BLI. (D-E) Binding capacity of n501-$(G_4S)_3$-αHSA, n501-$(G_4S)_4$-αHSA and their cysteine mutation variants to the HSA and MSA antigen, as measured by ELISA.

Figure S2

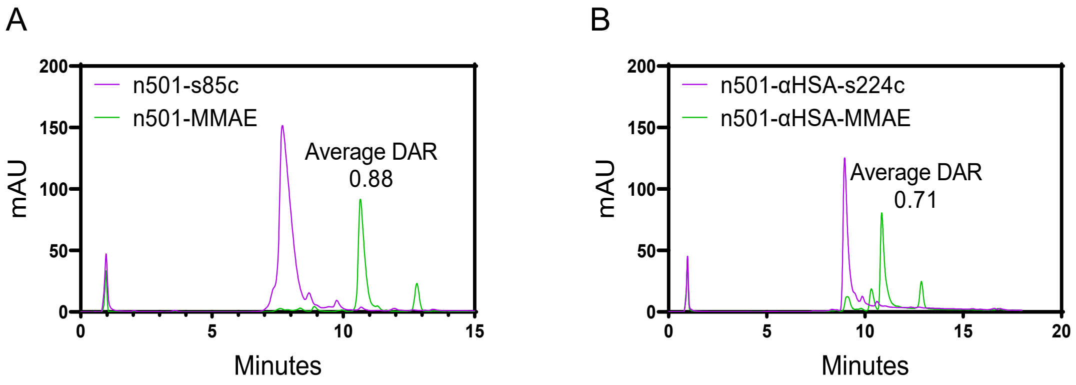

Figure S2. The average DAR was measured by RP-HPLC (reversed-phase high performance liquid chromatography).

Representative RP-HPLC trace of (A) n501-s85c, n501-MMAE, and (B) n501-αHSA-s224c, n501-αHSA-MMAE.

Figure S3

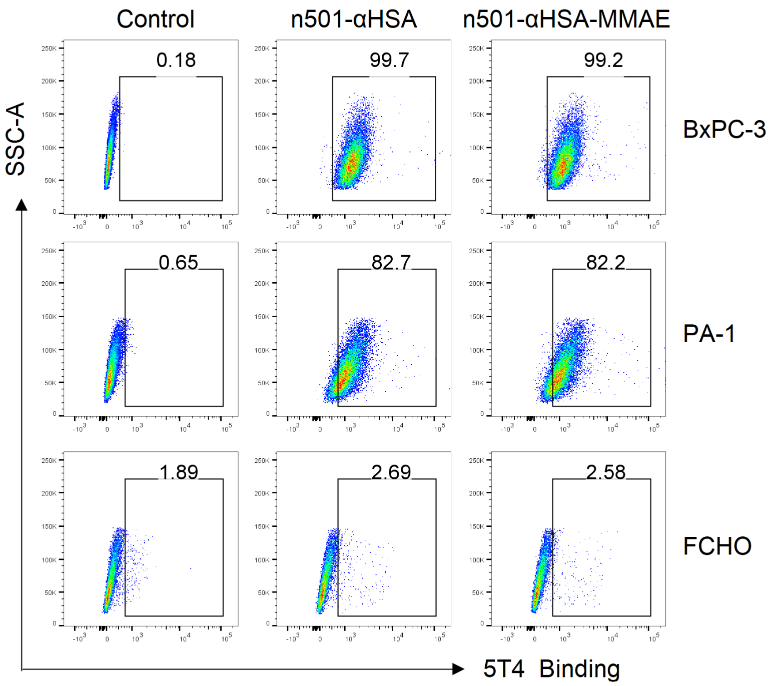

Figure S3. Binding specificity of n501-αHSA-MMAE to 5T4$^{+/-}$ cells

Figure S4

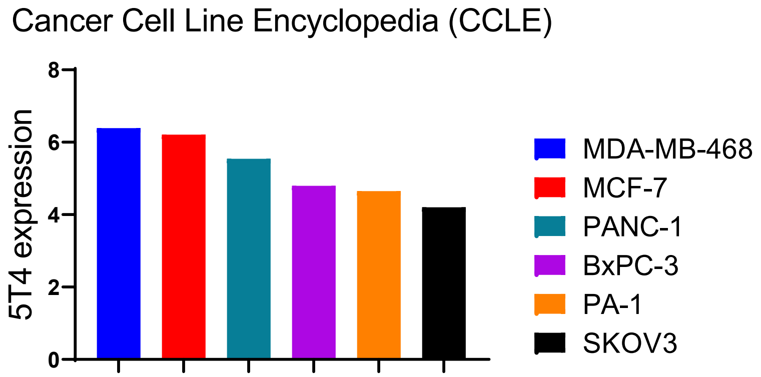

Figure S4. Statistics on the expression of 5T4 in different cell lines from the Cancer Cell Line Encyclopedia (CCLE) database (https://sites.broadinstitute.org/ccle//)

Figure S5

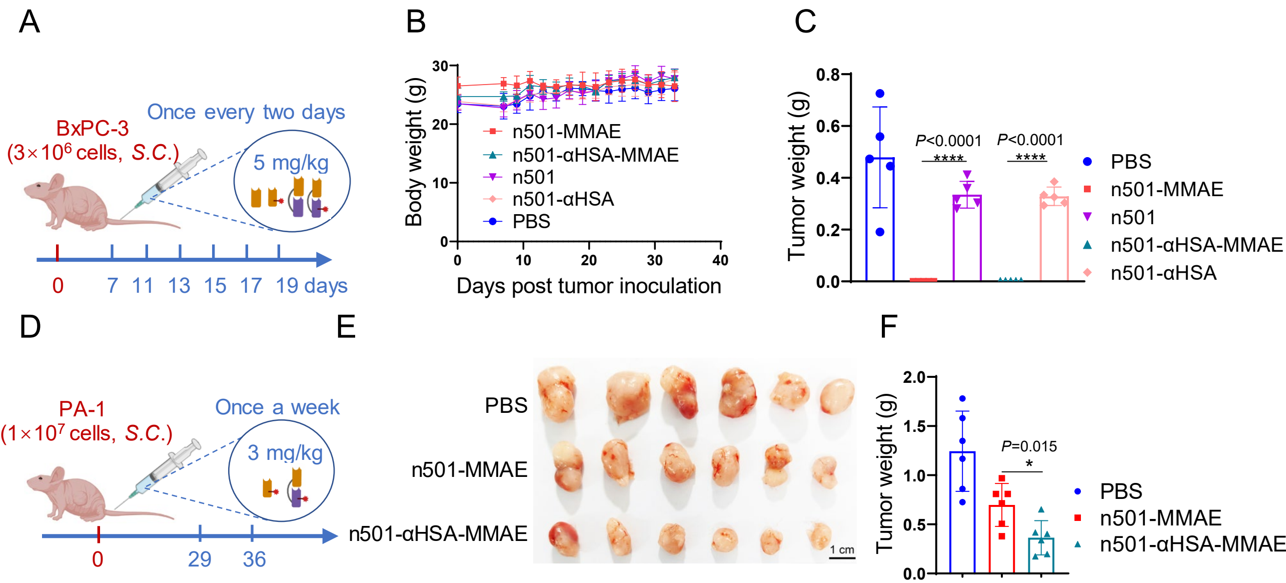

Figure S5. Therapeutic efficacy of n501-αHSA-MMAE in BxPC-3 and PA-1 xenograft mouse model.

(A) Schematic representation of in vivo experiments in BxPC-3 mouse model. (B) Mice weight were detected after tumor inoculation. (C) After 33 days post tumor inoculation, the tumor of the mouse was peeled off and weighed (n=5). (D) Schematic representation of in vivo experiments in PA-1 mouse model. (E-F) After 39 days post tumor inoculation, the tumor of the mouse was peeled off for photographing and weighing (n=6).

Figure S6

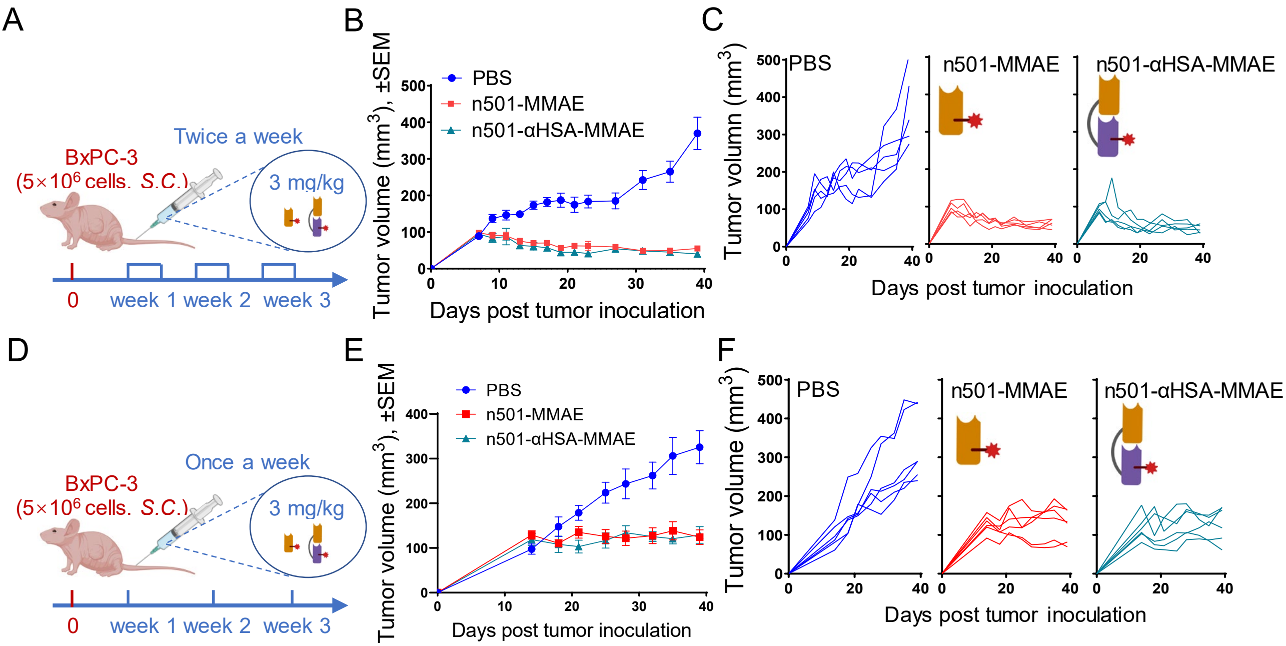

Figure S6. Comparison of the therapeutic effects of n501-MMAE and n501-αHSA-MMAE in BxPC-3 xenograft mouse model.

(A) Schematic representation of in vivo experiments in BxPC-3 mouse model. BxPC-3 xenograft mice model was treated 3 mg/kg n501-MMAE and n501-αHSA-MMAE intravenously for 6 times twice a week. (B-C) the average tumor volume (mm^3) (B) and individual tumor volume (C) were measured (n=5). (D) Schematic representation of in vivo experiments in BxPC-3 mouse model. BxPC-3 xenograft mice model was treated 3 mg/kg n501-MMAE and n501-αHSA-MMAE intravenously for 3 times once a week. (E-F) The average tumor volume (mm^3) (E) and individual tumor volume (F) were measured (n=6).

Figure S7

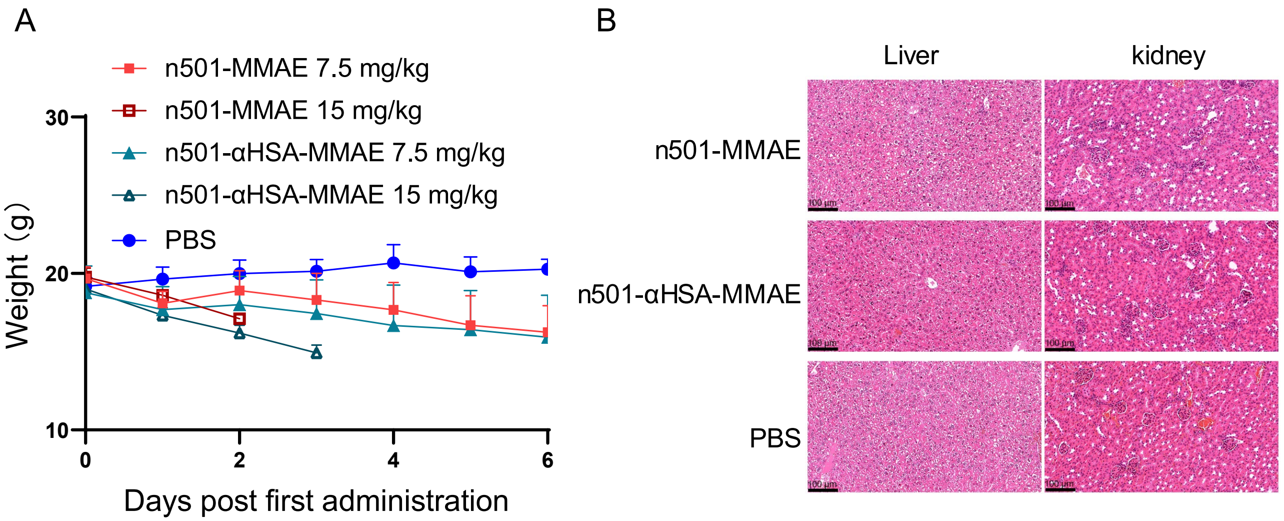

Figure S7. (A) Schematic of treatment regimen. Mice were treated with 7.5 mg/kg or 15 mg/kg drugs daily for 6 doses. The body weight of mice was measured. (B) Histopathological examination (H&E staining, ×200) of liver and kidney of BALB/c mice treated with 7.5 mg/kg n501-MMAE and n501-αHSA-MMAE.
